# Septic Arthritis of the Pediatric Shoulder: From Infancy to Adolescence

**DOI:** 10.1155/2016/3086019

**Published:** 2016-08-21

**Authors:** Justin W. Walker, William L. Hennrikus

**Affiliations:** ^1^College of Medicine, The Pennsylvania State University, Hershey, PA 17033, USA; ^2^Penn State Hershey Medical Center, Bone and Joint Institution, Hershey, PA 17033, USA

## Abstract

*Background*. Septic arthritis of the glenohumeral joint in the pediatric population (<18 yo) is not commonly described in the literature. There is a corresponding paucity of information regarding its presentation and treatment.* Methods*. An IRB approved review of patients treated with irrigation and debridement by the Orthopaedic Department was completed. This retrospective study includes four patients, presenting from 2005 to 2015, with septic arthritis of the shoulder.* Results*. The mean age (*M*_age_) at presentation was 5 years, with a range of 1 month to 15 years. Patients presented on average after 7 days with pain and a mean temperature of 39°C, erythrocyte sedimentation rate of 66 mm/hr, a C-reactive protein level of 11.17 g/dL, and a white blood cell count of 20.2 × 10^3^/mcL.* Staphylococcus aureus*,* Candida albicans*, and* Pseudomonas aeruginosa* were cultured from the wounds. All cases were treated operatively with irrigation and debridement and with antimicrobial therapy. Patients received antibiotics for an average of 6 weeks.* Conclusion*. Septic arthritis of the shoulder occurs in all pediatric ages. Successful treatment of septic arthritis of the shoulder was accomplished in four cases without division of the biceps sheath, with an average follow-up of 8 months.

## 1. Introduction

Septic arthritis of the pediatric shoulder is a rare condition, with most reports citing a prevalence of 3–5% of all septic joints [1]. As such, there is a lack of data available regarding its clinical presentation, clinical course, and treatment. Additionally, septic arthritis of the shoulder is often stated to be a disease of infancy and is commonly associated with osteomyelitis of the adjacent bone [1–3]. The anatomy of the shoulder joint likely contributes to the association with osteomyelitis, as the capsule of the shoulder envelopes the metaphysis, facilitating hematogenous spread between the bone and joint space [2]. Treatment recommendations vary; however, surgical opening and exploration of the glenohumeral capsule and biceps tendon sheath are recommended for thorough cleansing of the joint [4]. In this review, 42 children with septic joints were identified, of whom 4, or 9.5%, were found to have septic arthritis of the shoulder.

## 2. Materials and Methods

Following medical school Institutional Review Board (IRB) approval, review of all records, from 2008 to 2015, for pediatric patients (<18 yo) with septic shoulders treated with irrigation and debridement was completed. Forty-two patients with septic arthritis were identified, of whom 5 had an infected shoulder. One of the five was a 17-year-old female who acquired a shoulder infection as a postoperative complication and was excluded from the current study (*N* = 4). Special attention was paid to the time required for diagnosis, laboratory data, treatment strategies, and long-term complications.

## 3. Results

Seventy-five percent of patients in this study were male. The average age was 5 years with a range of 1 month to 15 years. The average time until diagnosis was 7 days, with a range of 4 to 10 days. All four patients presented with decreased use of the affected shoulder, and 1 of the 4 had an antecedent trauma (case 2). This patient fell onto the affected shoulder during a basketball game “a few weeks” prior to presentation. Two patients were born prematurely at 27 weeks (case 3) and 32 weeks (case 4). See Table 1 for a synopsis of cases.

Three of 4 patients had elevated C-reactive protein (CRP) levels on laboratory data with an average of 11.17 g/dL (0.8–14.3 g/dL). The erythrocyte sedimentation rate (ESR) was reported in 3 of the 4 patients, with an average of 66 mm/hr (20–125 mm/hr). The average temperature was 39.0°C. The white blood cell count (WBC) had an average of 20.19 × 10^3^/mcL (17,000–23,800).

A positive culture from synovial fluid was obtained in 100% of patients. These consisted of methicillin-resistant* Staphylococcus aureus* (two), methicillin-sensitive* Staphylococcus aureus*,* Candida albicans*, and* Pseudomonas aeruginosa*. One patient's culture grew both* Candida albicans* and methicillin-sensitive* Staphylococcus aureus.*

One patient was treated with an aspiration followed by arthrotomy and antibiotics, while the other three were treated with arthrotomy and antibiotics. In all surgeries, the biceps tendon sheath was left intact. Patients were treated with antibiotics for an average of 43 days, with a range of 32 to 48 days. In 3 of 4 cases, both parenteral and oral antibiotics were used. Empirical treatment with vancomycin was initiated in all four cases until culture returned and a more narrow-spectrum antibiotic could be chosen.

The average follow-up of these patients was 8 months, with a range of 2 months to 12 months. Of these patients, one reported decreased range of motion at 2 months of follow-up (case 1).

## 4. Discussion

Septic arthritis of the glenohumeral joint is considered by many to be a disease of infancy [1, 2, 5, 6]. However, in this study, only 2 patients were infants (*M*_age_ = 5 years). Each patient presented to the emergency department, three as transfers from an outside institution and one from home. In each case, the patient's chief complaint included pain with motion in the glenohumeral joint. In case 3, the patient also presented with decreased movement of the right lower extremity secondary to a concomitant knee infection.

The initial diagnosis of septic arthritis requires astute clinical judgment. Literature regarding the initial evaluation of children with joint pain or concern for septic arthritis recommends obtaining a full blood count with differential, ESR, CRP, synovial fluid analysis, and synovial fluid culture [6–9]. Of the laboratory studies done in this study, the most consistently elevated marker was total white blood cell count (>12,000), which was present in 100% of the cases. The ESR and CRP were elevated (>2 g/dL and >40 mm/hr, resp.) in 75% of the cases. However, the ESR was not requested in one of the cases, likely due to the patient's age (3 months). The elevated CRP in 75% of the cases supports recent literature recommending the inclusion of CRP in the diagnostic criteria for septic arthritis [8].

Cultures obtained from these cases were positive for MRSA (2), MSSA (1),* Pseudomonas aeruginosa* (1), and* Candida albicans* (1). One of the cases had coinfection with both MSSA and* C. albicans*. One of 4 patients was bacteremic, and another was both bacteremic and fungemia.

The fungemia patient, 4-month-old born at 27-week gestation, had multifocal septic arthritis, with positive cultures for* Candida albicans* and MSSA found in both the glenohumeral and acetabulofemoral joints. The severity of this presentation and pathogen involved can be explained partly by the increased susceptibility of infant's joints to hematogenous spread of infection, as well as the immature immune system associated with infants of preterm birth [5, 10, 11].

Fifty percent of cases in this review presented with concomitant osteomyelitis and septic arthritis of the shoulder. These data are similar to the reported rate of 75% by Montgomery et al., 67% by Belthur et al., and 56% by Schmidt et al. [3, 9, 12]. In a separate study, it was noted that 100% of patients (*N* = 6) with osteomyelitis of the proximal humerus had septic arthritis of the shoulder [4]. In all cases of osteomyelitis in the current study, the metaphysis was drilled in order to decompress the bone, in keeping with previous recommendations [2].

In addition to laboratory evaluation, imaging modalities are recommended in the evaluation of septic arthritis [1, 2, 4]. In all 4 cases, imaging modalities were used to assist in the diagnosis of septic arthritis, with MRI being the modality of choice. Initial plain radiographs were obtained in all four cases and failed to note abnormalities in 75% of the patients. In addition, plain radiograph had a false-negative rate of 50% for changes consistent with osteomyelitis. Technetium-pyrophosphate bone scans have been reported as useful in the literature, as they assist in the diagnosis of skip lesions in osteomyelitis, which are often missed when localized MRIs are used [13]. Bone scans were not used in the diagnosis of the patients in this series.

Treatment of septic arthritis of the shoulder includes appropriate antibiotic therapy and drainage. Infectious disease consult is prudent and was done in all 4 cases. Repeat aspiration and irrigation and debridement are reported modalities of drainage for septic arthritis of the shoulder [14]. However, one author recommends dividing and exploring the biceps tendon sheath to ensure full debridement of infected tissue [3]. In the current study, the surgical exploration and treatment left the biceps tendon intact, with a thorough irrigation of the joint noted in each procedure.

On follow-up, the patients' outcomes were generally good. None of the patients had long-term clinical deficits. The patient in case 2 was noted to have sclerotic changes to the proximal humerus on radiograph, without clinical manifestations of these changes. This patient was able to return to basketball at a collegiate level.

Previous studies demonstrated a delay in treatment for shoulder infection as compared to hip infections and postulated that the lack of an evidence-based clinical algorithm may contribute [9]. The Kocher criteria were developed as a joint-specific algorithm for the diagnosis of septic arthritis of the pediatric hip and have facilitated in the diagnosis of septic hips [15]. Joint insensitive clinical algorithms do exist for the diagnosis of septic arthritis [16, 17]. Sen et al. (2015) [7] recommend that patients with an acute joint in the outpatient setting receive routine lab work involving serum WBC, ESR, and CRP. Mathews and Coakley (2008) stated that though serum WBC, ESR, CRP, and temperature were all useful in the evaluation of an acute joint, they were not diagnostic [17]. Synovial white cell count is cited as being the only predictive laboratory value, with values ≥17,500 having sensitivity and specificity of 83% and 67%, respectively, and is considered not better than the “gold standard,” which is clinical suspicion by an expert physician with experience in musculoskeletal disease [16, 18]. Therefore, a more specific set of diagnostic criteria for septic shoulders and increased clinical suspicion would facilitate the clinician in making a prompt diagnosis and thus improve clinical outcomes [2, 3, 9, 10]. Additionally, it is important to note that the age of the patients within this study is markedly advanced in comparison to previous literature, and therefore clinicians should have appropriate suspicion for septic arthritis of the shoulder, even in the older child.

## 5. Summary

Septic arthritis of the shoulder is a rare condition. Although generally associated with infants, septic arthritis of the shoulder must be considered when evaluating any patient with shoulder pain and systemic signs of inflammation. We report four cases successfully treated with appropriate antibiotic use and surgical drainage. Septic arthritis of the shoulder may put patients at risk for long-term changes of the articular surface of the joint, and prompt diagnosis and management must be undertaken to reduce the severity of these complications. Development of diagnostic criteria specific to the shoulder would be facilitated in this endeavor and should be addressed in future literature.

## Figures and Tables

**Table 1:** Septic arthritis findings.

Case/age/sex	Time before treatment (days)	Temperature max. (°C)	Initial WBC (×10^3^/mcL)	Initial ESR (mm/hr)	Initial CRP (mg/L)	Initial X-ray findings	Culture results	Treatment	Associated problems	Complications/follow-up
2/3 y/M	7	39.4	23.8	125	14.3	Negative	Methicillin-resistant *S. aureus*	Arthrotomy, antibiotics 48 days	None	Decreased range of motion/2 mo
3/15 y/M	10	40.9	19.89	53	27	Negative	Methicillin-resistant *S. aureus*	Aspiration, arthrotomy, antibiotics 32 days	Multifocal osteomyelitis, septic knee, bacteremia	None/12 mo
4/8 mo/F	7	38.1	20.1	N/A	2.56	Negative	*Candida albicans,* methicillin-sensitive *S. aureus*	Arthrotomy, antibiotics 48 days	Septic hip, premature, bacteremia, fungemia	None/9 mo
5/1 mo/M	4	37.7	17	20	0.8	Concern for osteomyelitis	*Pseudomonas aeruginosa*	Arthrotomy, antibiotics 43 days	Premature	None/7 mo

## Abbreviations

ESR: Erythrocyte sedimentation rate
WBC: White blood cell
CRP: C-reactive protein.
